# Decoding of coherent but not incoherent motion signals in early dorsal visual cortex

**DOI:** 10.1016/j.neuroimage.2010.04.011

**Published:** 2011-05-15

**Authors:** Dietrich Samuel Schwarzkopf, Philipp Sterzer, Geraint Rees

**Affiliations:** a UCL Institute of Cognitive Neuroscience, 17 Queen Square, London WC1N 3AR, UK; b Wellcome Trust Centre for Neuroimaging at UCL, 12 Queen Square, London WC1N 3BG, UK; c Department of Psychiatry, Charité Campus Mitte, Charitéplatz 1, D-10117 Berlin, Germany

**Keywords:** Coherent object motion, Functional brain imaging, Multivariate pattern decoding, Direction, Perceptual grouping

## Abstract

When several scattered grating elements are arranged in such a way that their directions of motion are consistent with a common path, observers perceive them as belonging to a globally coherent moving object. Here we investigated how this coherence changes the representation of motion signals in human visual cortex using functional magnetic resonance imaging (fMRI) and multivariate voxel pattern decoding, which have the potential to reveal how well a stimulus is encoded in different contexts. Only during globally coherent motion was it possible to reliably distinguish fMRI signals evoked by different directions of motion in early visual cortex. This effect was specific to the retinotopic representation of the visual field quadrant in V1 traversed by the coherent element path and could not simply be attributed to a general increase in signal strength. Decoding was more reliable for cortical areas corresponding to the lower visual field. Because some previous studies observed poorer speed discrimination when motion was grouped, we also conducted behavioural experiments to investigate this with our stimuli, but did not reveal a consistent relationship between coherence and perceived speed. Taken together, these data show that neuronal populations in early visual cortex represent information that could be used for interpreting motion signals as unified objects.

## Introduction

The detection and identification of partially occluded objects, such as spotting a predator or prey moving through foliage, is essential for survival. Our visual system has an almost effortless ability to extrapolate objects even when only limited information is available by grouping elements from different portions of the visual field. Despite its ubiquity, the processes by which the brain groups such simple but separated features to form percepts of coherent objects remains poorly understood.

Many psychophysical studies have explored how grouping affects perception. Detection thresholds are enhanced for targets presented in a coherent context (Kovacs and Julesz, 1994; Polat and Bonneh, 2000; Polat and Sagi, 1993). Observers are also more adept at finding smooth contours and shapes embedded in noisy environments than simple feedforward models of visual processing would predict (Field et al., 1993; Hess et al., 1997). Not all effects are facilitatory. Vernier acuity is reduced when a collinear stimulus is placed in between the two Vernier targets (Zhu and Liu, 2009). Moreover, observers are poorer at judging the speed of several moving stimuli that are perceived as being grouped relative to when they are perceived as being independent (Verghese and McKee, 2006; Verghese and Stone, 1996). Thus grouping can both facilitate and interfere with the processing of grouped features.

How is information about a coherent object encoded in the visual cortex? Neurophysiological studies on perceptual integration have resulted in conflicting results. Single-unit microelectrode recordings in animals suggest a role for early visual cortex in grouping segregated image features into a coherent global percept. For example, the firing of V1 neurons in response to oriented bars is modulated by the presence of flanking bars outside the classical receptive field (Gilbert, 1992; Kapadia et al., 2000) or when their receptive fields fall inside a segregated surface compared to a homogeneous field (Zipser et al., 1996). Neuroimaging studies in humans are inconsistent, showing both weaker (Fang et al., 2008; Harrison et al., 2007; Murray et al., 2002), and stronger responses for grouped or spatially coherent stimuli (Altmann et al., 2003; Schwarzkopf et al., 2009; Zhu and Liu, 2009). One possible explanation for this discrepancy is that V1 activity is driven by low level image statistics (Dumoulin and Hess, 2006). Reduced activity may also reflect a reduction in inhibitory neuronal activity, which obscures the selective facilitatory effects measured with single-unit recordings (Kinoshita et al., 2009).

**Fig. 1:**
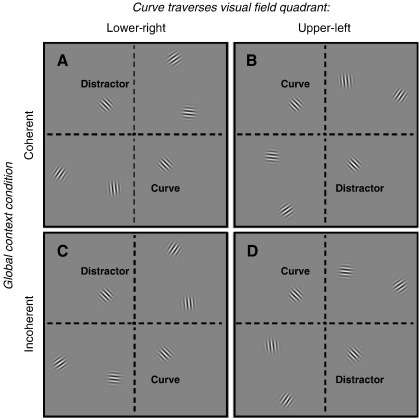
Static illustrations of the stimuli used. Five Gabors were placed on an imaginary curved path. Elements were either oriented in a coherent (A–B) or incoherent (C–D) relationship with the global context of the path. A sixth Gabor (the distractor element) was located in the diametrically opposite location to the middle element of the curve (the curve element). Within separate scanning sessions the individual elements making up the curve either traversed the lower-right (A, C) or the upper-left (B, D) visual field. Movies of the actual stimuli used in the experiment are included in the supplementary materials.

Previous research focused only on the level of activity rather than exploring qualitatively how grouping alters the encoding of a stimulus. We therefore set out to investigate this issue using high-resolution functional magnetic resonance imaging (fMRI) and multivariate voxel pattern decoding. Rather than merely establishing that a stimulus dimension is encoded within a brain region, the strength of multivariate voxel pattern decoding lies in its potential for examining how well a stimulus presented in different contexts is encoded in distributed responses across visual cortex (Haynes and Rees, 2005; Kamitani and Tong, 2005). For instance, it has been employed to study differences in conscious and unconscious stimulus representations (Haynes and Rees, 2005; Sterzer et al., 2008), or to infer the focus of feature-based attention (Kamitani and Tong, 2005, 2006). Here we explored how coherent motion altered the encoding of direction of motion in retinotopic visual cortex. We examined the phenomenon that occurs when only local parts of an object are visible, such as when an object moves behind occluding surfaces. This can be mimicked when several small drifting grating elements are arranged in such a way that the individual directions of motion are together consistent with a smooth path (Figs. 1A–B; and see the accompanying movies in the supplementary materials). Under these conditions, observers perceive all elements as part of a larger entity, while this is not the case for grating elements that are also positioned along a path but whose directions of motion are inconsistent with the path (Figs. 1C–D). This paradigm allowed us to examine how the representation of the motion of individual stimulus elements in early visual cortex varied depending on whether their motion was part of a larger coherent context. Since we presented elements in different visual quadrants, one element was separated from its context not only in visual space but in terms of its representation in distinct parts of retinotopic cortex compared to the context. Only the relationship between this element and the context (coherent versus incoherent) varied while the individual motion of the stimulus element itself remained constant. Thus any changes in spatial patterns of blood oxygen level dependent (BOLD) signals evoked by this element in visual cortex must reflect the effect of such global coherence rather than changes in the individual stimulus elements.

To anticipate our findings, we showed that only when elements are spatially coherent was it possible to decode the direction of a moving grating element from voxel response patterns in early visual cortex. Decoding was only reliable in regions corresponding to the lower visual field compared to the upper visual field. Because previous behavioural studies reported that motion grouping impaired speed discrimination (Verghese and McKee, 2006; Verghese and Stone, 1996), we also performed behavioural experiments outside the scanner to test whether our observed changes in the neural representation of motion are involved in this process. We did not reveal a consistent relationship between coherent motion and perceived speed.

## Materials and methods

### Functional imaging experiment

#### Participants

Nine healthy participants (4 females, 2 left-handed, age: 20–33) gave written informed consent to participate in the experiment, which was approved by the local ethics committee. Participants were naïve to the purpose of the experiment, except for one of the authors (DSS), and had normal or corrected-to-normal vision.

#### Stimuli

During fMRI scanning, participants viewed movies comprising several drifting Gabor elements, i.e. sinusoidal carrier gratings (spatial frequency: 2.7 cycles/°) convolved with Gaussian apertures (standard deviation: 0.55°), in which drift was induced by advancing the phase of the carrier by 30° on each video frame (i.e. they drifted at 5 cycles/s). Each stimulus movie always comprised six Gabor elements, five of which were positioned on a curved imaginary path (160° sector of a circular path) that arched through three quadrants of the visual field (eccentricities: 4.1°–8.4°). The elements on this path were separated by 5.5° of visual angle. There were two types of stimuli that differed in their global context, coherent and incoherent. In the coherent context the orientation of the Gabors was orthogonal to the path and thus their motion trajectories were tangential to it. We generated the incoherent context by swapping the orientations of the elements flanking the curve element (that is, e.g. the outer element in the upper visual field was swapped with the outer element in the lower visual field). This ensured that the difference in orientation/direction between neighbouring elements was similar to the coherent stimuli, but that the motion was no longer consistent with the curved path. Crucially, the middle element of the context (curve element) was identical for both the coherent and incoherent contexts. Moreover, the middle element was located in a different visual field quadrant than the nearest context element (Fig. 1). As individual quadrants can be reliably separated in early retinotopic cortex, activity related to the middle element could thus be dissociated from activity evoked by the context elements. The sixth Gabor element (distractor element) was positioned in the visual field quadrant diagonally opposite the curve element at 4.1° from the centre of gaze. The distractor element always had the same orientation as the curve element, but to prevent the distractor from being grouped with the global context, the direction of motion of the distractor was always opposite to that of the curve element.

The global context (that is the curved path) always spanned three visual field quadrants between the lower-left and upper-right quadrants. However, it could curve either through the lower-right (Figs. 1A, C) or the upper-left quadrant (Figs. 1B, D). Within any one fMRI session the location of the path was constant. A small black fixation cross was present at all times. Stimuli were back-projected onto a screen participants viewed via a front-surface mirror attached to the headcoil. The stimuli are illustrated in Fig. 1 and examples of the movies can be viewed in the supplementary information. Stimuli were generated in MATLAB (Mathworks) and presented using the Cogent toolbox (http://www.vislab.ucl.ac.uk/cogent.php).

#### Procedure

In each scanning run, participants viewed continuous movies of the four stimulus conditions, coherent and incoherent, with anti-clockwise or clockwise motion, respectively. Each condition was presented twice, in a pseudo-randomized order (with the constraint that the same condition could never be presented twice in a row). Movies lasted 19.2 s and were interleaved with 19.2 s blank periods during which only the fixation cross was being presented. In order to ensure fixation and maintain arousal, the participants were required to monitor the fixation cross for a small increase in luminance to which they responded by pressing a button on a MRI-compatible keypad. These events were 200 ms in duration and occurred with a probability of 0.05 every 400 ms throughout the run. Altogether, there were 8 scanning runs of the main experiment per session.

In addition to the main experiment, during one of the scanning sessions participants also participated in a number of different ‘localiser’ scans. First, an ‘element localiser’ was used to define retinotopic representations of the individual grating elements in early visual cortex. Participants viewed contrast-reversing (4 Hz) checkerboard elements of the same physical dimensions as the Gabor elements in the main experiment, alternating between the positions of the distractor and curve elements of the main experiment. The flickering elements were presented in 8 blocks of 25.6 s interleaved by 9.6 s fixation periods. In a second ‘motion localiser’ scan, participants viewed random dot stimuli consisting of 2000 dots (equal number of black and white dots) presented around fixation within a circular aperture (radius: 11.5°). Stimulus conditions were similar to the ones described in Dupont et al. (1997): (1) static dots, (2) transparent motion in which half the dots moved in opposite directions and (3) kinetic contours in which bands (width: 2.3°) moved in opposite directions. For stimuli containing motion the direction of motion changed every 800 ms (directions from 0 to 345° with 15° increments). For the static stimuli, a new random dot stimulus was presented every 800 ms. Finally, we also collected fMRI data for retinotopic mapping showing 10 cycles (duration: 38.4 s) of a smoothly rotating contrast-reversing (4 Hz) checkerboard wedge.

#### Data acquisition

Functional data were acquired on a 3 T Allegra head scanner (Siemens Medical Systems, Erlangen, Germany), using a standard transmit–receive head coil with a single-shot gradient echo isotropic high-resolution EPI sequence (matrix size: 128 × 128; FOV: 192 × 192 mm^2^; in-plane resolution: 1.5 × 1.5 mm^2^; 32 oblique transverse slices with interleaved acquisition; slice thickness: 1.5 mm, no gap; TE: 30 ms; acquisition time per slice: 100 ms; TR: 3200 ms; echo spacing: 560 µs; receiver bandwidth: 250 kHz; 30% ramp sampling; 2-fold read oversampling to allow for *k*-space re-gridding; read gradient amplitude: 34.47 mT/m; read gradient slew rate: 344.7 mT/m/ms; flip angle *α* = 90°). Slices were angled at 30° to maximize coverage of the calcarine sulcus and the occipital lobes. For two participants we measured eye movements and pupil diameter (continuously sampled at 60 Hz) during scanning using a video eye tracker (ASL 504LRO Eye Tracking System, Mass).

In each scanning run in the main experiment and the motion localiser we acquired 108 volumes, in the ‘element localiser’ 97 volumes and in the retinotopic mapping run 130 volumes. The first six volumes were removed from any subsequent analysis to allow for T1 equilibration. To correct for EPI distortions induced by susceptibility artifacts, we acquired double echo FLASH images to estimate maps of the *B*_0_ field. Finally, we acquired T1-weighted anatomical images using a MDEFT sequence.

#### Initial data analysis

Neuroimaging data were preprocessed and analysed using SPM5 (http://www.fil.ion.ucl.ac.uk/spm). Functional images were corrected for slice acquisition time, realigned to the first image using an affine transformation to correct for small head movements and EPI distortions unwarped using *B*_0_ field maps (Hutton et al., 2002). Data were smoothed with a Gaussian kernel with 5 mm FWHM. The resulting images were entered into a participant-specific general linear model with conditions of interest corresponding to each category of visual stimuli. Blocks were convolved with a canonical haemodynamic response function to generate regressors. In addition, the estimated head movement parameters were entered as regressors of no interest. Linear contrasts among the condition-specific regressors were used to identify regions of interest in the localiser scans.

#### Delineation of visual areas

From the anatomical images we reconstructed and inflated the surface of each cortical hemisphere using FreeSurfer (http://surfer.nmr.mgh.harvard.edu/fswiki) (Dale et al., 1999). Polar maps of the visual cortex were calculated using phase-encoded retinotopic mapping techniques (Sereno et al., 1995) and retinotopic visual areas were delineated manually. The boundaries of V1–V3 were delineated by identifying the representation of the vertical and horizontal meridians from the mirror reversals in the phase map, separating the ventral and dorsal subregions of these areas. V4 and V3A were defined as maps of a full visual hemifield, anterior to V3v and V3d, respectively. We also mapped regions of interest (ROI) for the activations in the element localiser in left dorsal V1 (lower-right element) and right ventral V1 (upper-left element). We identified the motion-selective V5/MT complex by the linear contrast between transparent motion stimuli and the static random dot stimuli. Further, we mapped a region of interest anterior to area V3A in the lateral occipital cortex that was activated preferentially by kinetic boundaries relative to the transparent motion. We refer to this region as KO (‘kinetic occipital’) (Dupont et al., 1997), although this encompasses several retinotopic areas and parts of the lateral occipital complex (Larsson and Heeger, 2006).

#### Multivariate voxel pattern decoding

Preprocessed functional data in volume space were further analysed using custom software written in MATLAB. The time course from each run was *z*-score normalized. The voxels belonging to each ROI were identified by projecting the labelled surface vertices back into voxel space. For each ROI the data of voxels in each volume (shifted by 1 volume = 3.2 s to account for the lag of the haemodynamic response) were extracted and vectorized. Volumes from the same stimulus block were averaged so that there was only one voxel pattern (henceforth ‘samples’) for each block.

These data were then used for multivariate voxel pattern decoding using a leave-one-run-out cross-validation procedure, i.e. samples from all except one run were assigned to a training set and the remaining samples were used as a test set. For each condition we calculated the mean sample across all blocks in the training set. To decode we then calculated a linear correlation between each sample in the test set and the mean samples from the training set. A test sample was then assigned to the condition which produced the greater correlation coefficient. Decoding performance for each cross-validation was estimated as the percentage of correct classifications, and the final decoding accuracy was calculated by averaging performances from all eight cross-validations.

Since we used high-resolution fMRI, each ROI contained hundreds to thousands of voxels. We employed spatial smoothing with a narrow Gaussian kernel of 5 mm FWHM. The spatial pattern information exploited by many multivariate pattern decoding analyses is represented on a relatively coarse spatial scale (Gardner, 2010; Gardner et al., 2008; Kriegeskorte et al., 2010; Shmuel et al., 2010; Swisher et al., 2010) and spatial smoothing in this way does not diminish and can even improve decoding performance (Op de Beeck, 2010). We calculated the *t*-statistic for comparing the two conditions of interest only on the training data set, and then selected the most discriminative voxels by ranking voxels in both training and test sets in descending order (ignoring the sign of the *t*-statistic). Because the univariate voxel-wise difference was calculated only on the training data this ensured that the test data were unbiased. While this “nearest neighbour by correlation” method is arguably simpler than other more sophisticated machine learning algorithms and since the use of a linear SVM did not afford an increase in decoding accuracy we therefore chose a simple method for our analysis.

When decoding the type of context we grouped the samples from the clockwise and anti-clockwise motion from a particular context into a data set for one condition. This means that for this analysis there were twice the number of samples as for decoding the direction of motion, which was conducted separately for the coherent and incoherent contexts.

For most comparisons, the decoding accuracy obtained with the first 150 voxels was used for further statistical analysis, except for the decoding from the small element ROIs for which a cut-off of 28 voxels was used instead. These cut-offs were chosen by taking the minimal size of the ROIs across all participants.

### Behavioural experiment

#### Participants

Four participants, all of whom had participated in the functional imaging experiment, were recruited for a subsequent behavioural experiment (2 females, all right-handed, age: 27–30). Except for one of the authors (DSS) all participants were naïve to the purpose of this experiment. Participants had normal or corrected-to-normal vision and gave written informed consent for the experiment, which had been approved by the local ethics committee.

#### Stimuli

The stimuli used were the same as in the functional imaging experiment, except that we removed the distractor element so that only the elements on the path were visible. Moreover, in an additional condition only the curve element was presented without its neighbours.

#### Procedure

Participants took part in three sessions containing 630 trials each. We monitored eye movements using a high temporal-resolution (250 Hz) eye tracker (Cambridge Research Systems). Each trial was initiated when participants fixated on a small black fixation cross for a period of 600 ms. Subsequently, they viewed two 500 ms intervals with stimulus movies separated by a 500 ms blank period during which only the fixation cross was presented. If eye movements strayed from a circular window with 1.25° diameter around fixation, a red-circle appeared on the screen as feedback to the participant and the trial was discarded from any further analysis.

The participants' task was to decide which of the two stimulus movies was moving faster. One interval always contained only the curve element moving at the reference speed, which was varied on every trial (2.5–4.2 cycles/°). In the other interval, the test stimulus, Gabors moved at one of seven speeds relative to the reference speed (including a condition in which they were equal). The stimulus could either be only the curve element, the coherent context or the incoherent context. The direction of motion (anti-clockwise and clockwise) was chosen at random on every trial but was the same in both intervals. The order of the intervals was randomized. In half the trials the curve element was positioned in the lower-right or the upper-left visual field quadrant. All experimental conditions (i.e. stimulus configuration, visual field location and relative speed) were presented in a randomly interleaved order.

#### Data analysis

Psychophysical performance at the speed discrimination task was measured as the proportion of trials in which participants judged the test interval to be moving faster at each relative speed level. The raw data were then fitted with a logistic function using the psignifit toolbox (http://bootstrap-software.org/psignifit) implementing a maximum-likelihood procedure (Wichmann and Hill, 2001a,b). From the fitted psychometric functions we extrapolated the point of subjective equality (PSE), i.e. the relative speed at which participants believed the speed of the test stimulus to be equal to the reference.

## Results

### Decoding the global context: coherent versus incoherent

To investigate how object motion that was coherent across the individual elements (other than the distractor) altered the representation of motion in early visual cortex we measured fMRI blood oxygen level dependent (BOLD) signals while participants viewed movies comprising drifting Gabors (Fig. 1). The individual Gabor elements were either arranged so that their local motion was consistent with a global context of a smooth curve (coherent) or had their directions of motion scrambled (incoherent). First, we tested if it is possible to decode from voxel response patterns in early visual areas whether the global context was coherent or incoherent.

We used linear correlation for decoding by first calculating the average pattern for each condition from the samples in the training set, and then correlating these average patterns with each sample in the test set. Each test sample was assigned to the condition which produced the stronger correlation. Before decoding, data were spatially smoothed with a 5 mm FWHM Gaussian kernel in order to enhance the true pattern of voxel biases while reducing high frequency measurement noise. Qualitatively similar, albeit lower, decoding performance was obtained when unsmoothed data were used.

**Fig. 2:**
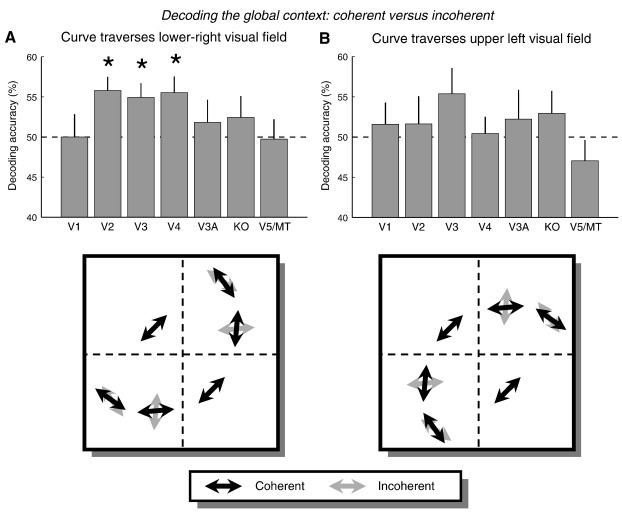
Discriminating the coherent versus incoherent contexts using voxel patterns from visual areas. Decoding accuracy averaged across participants is depicted for each ROI separately for when the curve traversed the lower-right visual field (A) or the upper-left visual field (B). Error bars denote ± 1 standard error of the mean. Asterisks indicate accuracy was significantly higher than chance (one-tailed *t*-test, *p* < 0.05). The arrows in the stimulus schematics indicate the directions of motion. Black arrows: coherent context. Grey arrows: incoherent context.

Fig. 2 plots the accuracy averaged across participants when decoding the global context (coherent versus incoherent) for visual areas. For V1–V4 and V3A this included all voxels from each area as delineated by the retinotopic mapping scan. For the higher extrastriate areas, i.e. KO and the V5/MT complex, the ROIs included all voxels which in the ‘motion localiser’ scan responded significantly (*p* < 0.05, uncorrected) more to kinetic contours than transparent motion (KO) or more to transparent motion than to static stimuli (V5/MT). This showed that when the curve traversed the lower-right visual field (Fig. 2A), accuracy for V2, V3, and V4 was greater than chance. These findings are consistent with the explanation that neuronal populations in higher extrastriate areas respond to the presence of a coherently moving object.

### No decoding of direction of motion for individual elements

The main purpose of our experiment was to examine how the presence of globally coherent motion changed the neural representation of motion associated with the retinotopic location of an individual Gabor element. In separate blocks the Gabors moved in opposite directions (anti-clockwise or clockwise with respect to the curved context). We reasoned that if perceptual grouping altered the representation of motion signals in early visual cortex, it should also affect the accuracy with which we could decode the direction of motion from spatially distributed voxel patterns in early visual cortex. Our stimulus design ensured that the physical stimulation in the lower-right and upper-left visual field quadrants was identical for the coherent and incoherent contexts. Therefore, on the basis of an independent ‘element localiser’ scan we defined retinotopic regions of interest in V1 corresponding to the Gabor elements in the lower-right and upper-left quadrants, and conducted our decoding analysis on voxels from these ROIs. It was impossible to decode direction of motion better than chance from voxels in the curve element regardless of retinotopic position (lower-right: *t*(8) = 0.33, *p* = 0.373; upper-left: *t*(8) = − 0.70, *p* = 0.748). Because it could be argued that spatial smoothing blurred the high spatial frequency patterns in these small ROIs, we conducted this analysis on unsmoothed data. However, for unsmoothed data performance was also consistently at chance (lower-right: *t*(8) = − 0.76, *p* = 0.765; upper-left: *t*(8) = − 0.66, *p* = 0.737). Thus, when restricting the analysis to the individual stimulus elements, it was not possible to reliably discriminate response patterns to the two directions of motion, in either global contexts.

### Decoding direction of motion from visual field quadrants

Next, we tested if there were contextual effects when also including parts of retinotopic visual cortex that did not represent parts of the visual field where our stimuli were presented. We performed the same decoding analysis for direction of motion on ROIs containing the entire quarter-field representations in early visual cortex, which received identical physical stimulation in the coherent and incoherent contexts. Thus we compared the decoding for ROIs representing the lower-right and upper-left visual fields. Henceforth, we will refer to the ROIs representing the visual field quadrants containing the curve and distractor elements as the ‘curve quadrant’ and ‘distractor quadrant,’ respectively (see Fig. 1).

**Fig. 3:**
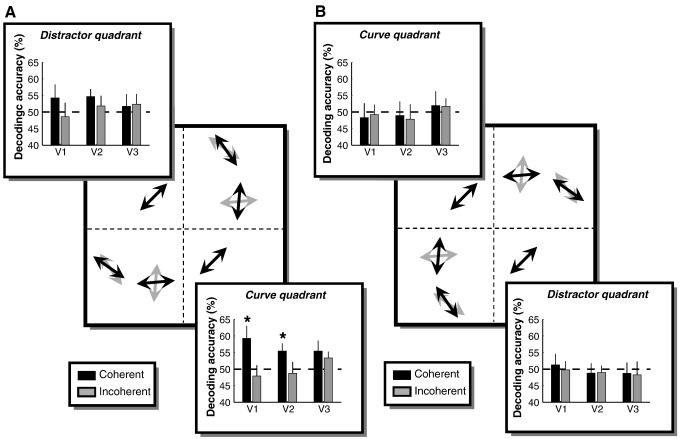
Decoding the direction of motion from voxel patterns in human visual cortex. Accuracy averaged across participants is plotted for retinotopic areas V1–V3 (error bars denote ± 1 standard error of the mean). The plots are superimposed on a stimulus schematic to indicate the ROI (i.e. the curve quadrant traversed by the global context or the distractor quadrant in the opposite hemifield). In separate experiments, the curve quadrant was either the lower-right (A) or the upper-left (B) visual field. Black arrows/bars: coherent context. Grey arrows/bars: incoherent context.

Fig. 3 depicts the decoding accuracy for each condition in areas V1–V3. We tested if decoding direction of motion was reliable, that is whether it was significantly greater than chance levels. In the curve quadrant in V1 decoding was better than chance for the coherent context (*t*(8) = 2.42, *p* = 0.020) but not for the incoherent context (*t*(8) = − 0.66, *p* = 0.737). Again, this difference was only significant when the curve quadrant was the lower-right quadrant; when it was the upper-left quadrant this difference was not significant (coherent: *t*(8) = − 0.40, *p* = 0.651; incoherent: *t*(8) = − 0.27, *p* = 0.601). At the level of individual participants we found that decoding of direction for the coherent context when the curve quadrant was the lower-right was significantly greater than chance in five out of the nine participants, while it was numerically greater than chance in another two. Conversely, when the curve quadrant was the upper-left only one participant showed above chance performance. Finally, decoding for the distractor quadrant was never significantly greater than chance. Taken together, our decoding results indicate that during grouping unstimulated parts of early retinotopic cortex contain information about the direction of motion of the grouped stimulus elements.

### The nature of the neural representation

The curve quadrant contained only the curve element, which remained constant between the coherent and incoherent contexts. The visual field quadrants are represented by spatially distinct regions of retinotopic cortex. Moreover, the population receptive field size of voxels in these regions and at the eccentricity of our stimulus is well below the separation of our Gabor elements (Dumoulin and Wandell, 2008; Smith et al., 2001). Although it is thus highly likely that the curve quadrant received physical stimulation from the visual quadrants containing the remaining elements on the curve, it cannot be ruled out completely that voxels in the curve quadrant might receive some input from the neighbouring quadrant representations. Moreover, it could be argued that voxels outside the ROI defined by the element localizer still showed a weak response to the experimental stimulus containing the full context.

**Fig. S1:**
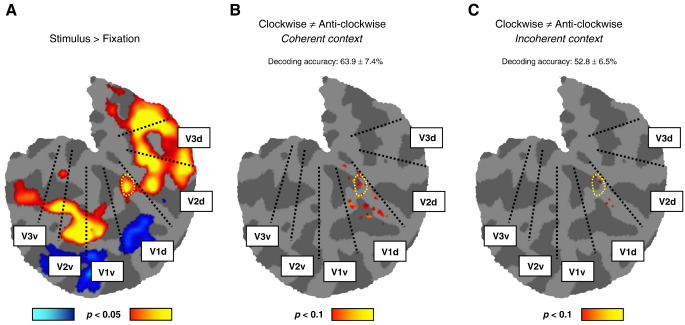
Flat maps from a representative participant. A. The response to all stimuli in the main experiment relative to blocks of fixation is plotted at a relaxed statistical threshold (*p* < 0.05, uncorrected). B–C. Voxels in V1d (representing the curve quadrant) showing a moderately significant bias for direction of motion (*p* < 0.1, uncorrected) in the coherent context (B) and the incoherent context (C). The dotted black lines indicate the boundaries of visual areas. The yellow dotted line denotes the location of the ROI defined by the ‘element localizer’. The mean decoding accuracy (± standard error of the mean) for direction of motion in V1d across all instances of cross-validation is denoted above each map.

In Fig. S1 we show maps of a representative participant on a reconstructed, inflated and flattened pial surface of the left occipital cortex. In these maps, V1d and V2d represent the curve quadrant, while V1v and V2v contain a map of the upper-right visual field, in which two of the inducer elements were presented. It is evident that even at a very relaxed statistical threshold (*p* < 0.05, uncorrected) the response to the stimulus in V1d does not dramatically exceed the ROI defined by means of the element localizer (yellow dotted line). More importantly, the activation evoked by the inducer elements in the context in V1v is distant from the response to the curve patch. Importantly, these maps are constructed from spatially smoothed data, and smoothing could conceivably have increased correlations between signals in voxels that encoded nearby locations in retinotopic space. However, since the smoothing kernel was small (5 mm FWHM), we observed that consistently good separation was achieved between the regions responding to the curve element and the inducers, respectively.

Improved decoding of the direction of coherent versus incoherent motion could be explained by a change of the signal-to-noise ratio of the pattern of voxel biases. We therefore calculated the absolute *t*-value for comparing clockwise and anti-clockwise motion across all runs in the data set, separately for the coherent and incoherent contexts. We reasoned that an increased signal-to-noise ratio would be reflected in a greater proportion of significantly biased (*p* < 0.05 on a *t*-test comparing response to clockwise and anti-clockwise motion) voxels for the coherent context. This revealed a trend towards significance in the effect of coherence (*F*(1,8) = 3.84, *p* = 0.086), providing some tentative evidence that indeed the voxels may have been more biased during coherent than incoherent motion. In the flat maps in Fig. S1 we plotted the absolute *t*-values for comparing clockwise and anti-clockwise motion for the coherent and incoherent context of V1d (representing the curve quadrant) in one participant. This map illustrates that when motion was coherent, more voxels exhibited a bias for the direction of motion, which resulted in above chance decoding for the coherent context only.

### Control analyses

The interpretation of our results requires that participants maintained accurate fixation throughout this experiment. While viewing the drifting Gabor stimuli they were asked to fixate on a small cross and press a response button whenever the cross changed luminance. Moreover, for two participants we tracked eye movements during the scan. Both measures indicate that participants maintained good fixation and were attentive (hit rate: 71.8%; see Supplementary information for full details of analyses).

**Fig. 4:**
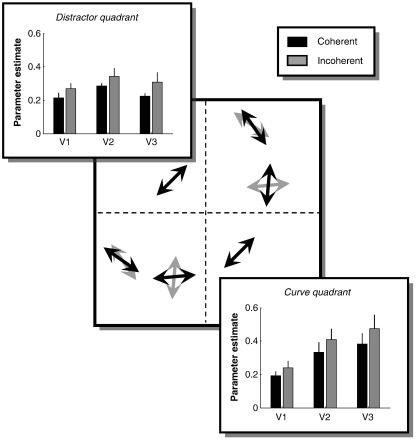
Mean BOLD signals evoked in early visual cortex by the globally coherent and incoherent contexts. Mean parameter estimates from the GLM analysis of all visually responsive voxels (see Materials and methods; averaged across participants) are plotted for regions of interest in early visual cortex. Panels are superimposed on a stimulus schematic indicating the ROI (curve quadrant and distractor quadrant). Only data from experiments when the curve quadrant was the lower-right visual field are shown. Black bars: coherent context. Grey bars: incoherent context. Error bars denote ± 1 standard error of the mean.

We also tested whether our MVPA results could be attributed to the size of overall signal evoked by the coherent and incoherent contexts. We conducted standard univariate analyses using a general linear model to estimate the overall activity evoked by each experimental condition within the quarter-field representations of retinotopic areas V1–V3 (Fig. 4). Because our multivariate voxel pattern decoding was only significantly better than chance for decoding direction of motion when the curve quadrant was the lower-right visual field we focused the conventional analysis also on this retinotopic location. A 3-way repeated measures ANOVA with the factors condition (global context: coherent and incoherent), quadrant (curve and distractor) and ROI (V1, V2 and V3) showed that there was a significant effect of ROI (*F*(2,16) = 13.24, *p* < 0.001), and a significant interaction between ROI and quadrant (*F*(2,16) = 5.83, *p* = 0.013). The response to the incoherent context was somewhat stronger than that to the coherent context, but the effect of context only showed a trend towards statistical significance (*F*(1,8) = 3.76, *p* = 0.088). We also compared the responses in retinotopic regions of interest within V1 that corresponded to the curve and distractor element, respectively, defined by means of the independent ‘element localiser’ scan. The difference between the coherent and incoherent contexts showed a trend towards significance (*F*(1,8) < 1, *p* = 0.071). There was no significant difference in the activity between the two elements (*F*(1,8) < 1, *p* = 0.807), and no interaction between condition and quadrant (*F*(1,8) < 1, *p* = 0.711). Taken together, there was no increase in overall signal for the globally coherent context in the curve quadrant which would result in enhanced voxel biases. If anything, the response to the incoherent context was subtly greater than that to the coherent context. Therefore, our MVPA results were not trivially accounted for by a difference in signal strength between global context conditions.

### Behavioural measurement of perceived speed

Finally, we also conducted a psychophysical experiment outside the scanner to investigate whether the enhanced decoding we observed for globally coherent context in early visual cortex might have any consequences for behaviour (other than the obvious perceptual difference in coherent versus incoherent motion). Previous reports indicate that perceptual grouping of moving stimuli can interfere with speed judgements (Verghese and Stone, 1996). Using a two-interval forced choice design we now tested whether the changes in representation of motion signals in early visual cortex that we had observed with our stimuli was associated with a similar change in perceived speed. During a probe interval either a single Gabor element (control) was presented or coherent or incoherent stimuli similar to those in the fMRI experiment were shown (except that the distractor element had been removed). Participants were asked to judge the speed against that of a single drifting Gabor element presented during a reference interval.

**Fig. S2:**
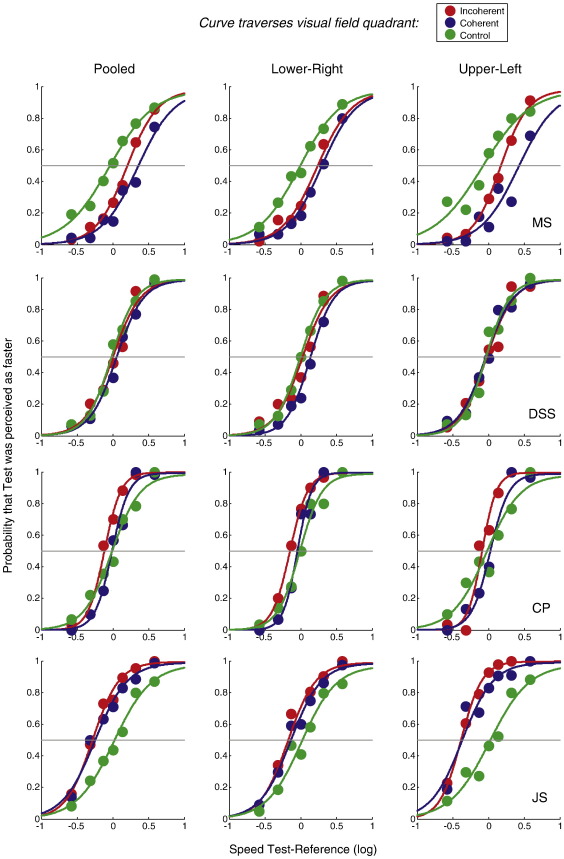
Psychometric curves in the behavioural experiment for each of the four participants (in rows). We plotted the proportion of trials in which the test stimulus was perceived as faster than the reference as a function of the veridical ratio between test and reference speed (in logarithmic units). Thus a rightward shift of the curve would indicate that participants saw the stimulus as moving more slowly than the reference stimulus. Data are shown either for pooling data from the two possible positions of the curve quadrant (left), or separately for when the curve quadrant was the lower-right (middle) or upper-left quadrant (right). Symbols indicate raw data; the solid lines are the best fitting models. Blue: coherent context. Red: incoherent context. Green: control (single element in both reference as well as test stimulus).

**Fig. 5:**
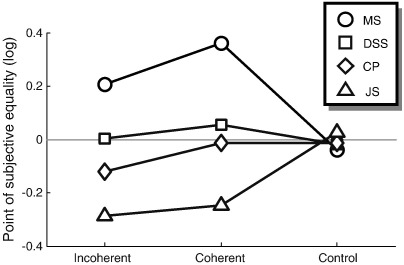
Behavioural experiment. The points of subjective equality for judging whether the Gabors moved faster than a reference element are plotted for each experimental condition and each of the four participants. Values indicate the ratio between the test and reference speeds (in logarithmic units). Thus, positive numbers indicate that participants saw the test interval as moving slower than the reference interval. Different symbols denote the data from individual participants. All data here are pooled across the two possible locations of the curve quadrant (lower-right and upper-left). Psychometric curves for individual observers and global context locations are shown in Fig. S2.

Fig. 5 shows the points of subjective equality for the four participants, i.e. the ratio (in logarithmic units) between test and reference speed at which participants judged the speed in both intervals to be equal. While participants showed a difference in their speed judgements between stimuli with several Gabor elements and the control condition with the single element, there was no consistent pattern of the bias across participants. While two participants (MS and DSS) perceived the speed of several elements to be slower than that of a single element, the other two participants (CP and JS) showed the opposite effect. Importantly, only participants MS and DSS perceived the coherent context to be slower than the incoherent context (the psychometric curves for each participant are plotted in Fig. S2).

## Discussion

Here we used functional MRI and multivariate voxel pattern decoding to investigate how motion signals in visual cortex were altered when the motion of individual elements scattered across the visual field was consistent with a coherent object. Participants viewed stimuli comprising drifting Gabor elements that were either aligned along a curved path or misaligned so that the local relationship between elements was preserved but globally motion was perceived as incoherent. We show that only for the coherent context presented to the lower hemifield was it possible to successfully decode the direction of motion of individual Gabor elements. We were unable to successfully decode direction for either context from voxels representing the upper hemifield. A relative lack of statistical power at the group level could conceivably account for our failure to find any significant decoding for the incoherent context or from any stimulus condition in the upper visual field. However, the same pattern of results was seen at the level of individual participants, with most showing significant decoding only for the coherent context when the curve quadrant was in the lower visual field.

Crucially, unlike previous neuroimaging studies on coherent motion, our use of multivariate pattern decoding went beyond a comparison of the level of responses evoked by coherent compared to incoherent motion. Rather, it allowed us to make a qualitative comparison of the neural representation of a stimulus feature (direction of motion). Our study thus contributes to our understanding of the neural processes through which retinotopically separated motion signals are interpreted as objects: when retinotopically separate stimulus features are consistent with the interpretation of a global entity, responses in the early visual cortex encode information about the unified object. Such contextual enhancement of motion signals may allow the visual system to extrapolate the movement of partially occluded objects. Because neurons representing the space between visible parts of an object are informative about the object's motion, they may also provide signals to infer the space the larger object occupies. This provides evidence that a coherent stimulus is encoded differently than just the sum of its individual component elements.

The present results are consistent with the finding that prolonged viewing of a perceptually grouped stimulus induces adaptation effects in parts of the visual field that do not receive direct physical stimulation (Roach et al., 2008). It is also in line with neuroimaging experiments showing that the size illusion caused by the three-dimensional context of a stimulus is accompanied by an enlarged retinotopic representation of the stimulus in V1 (Murray et al., 2006), by showing that other forms of contextual modulation also affect neuronal stimulus representations in human V1. Moreover, it also supports behavioural and electrophysiological evidence that contextual effects qualitatively alter the neuronal encoding of stimulus features in visual cortex (Kapadia et al., 2000).

### What underpins improved decoding?

Interestingly, we only observed reliable decoding of direction of motion of a single grating element when taking voxels from the entire visual field quadrant containing the element that belonged to the globally coherent stimulus. On the other hand, when we restricted our analysis to the retinotopic regions of interest corresponding to the stimulus elements, decoding was not significantly above chance. One possibility is that the ROI for individual elements may have contained too few voxels to permit reliable decoding, because elements were relatively small (standard deviation: 0.55°). We employed high spatial resolution fMRI with a voxel size of 1.5 × 1.5 × 1.5 mm^3^. With these parameters for most participants the ROIs for a single stimulus element contained only 50 voxels that may not have contained a sufficient number of biased voxels to result in reliable decoding. This possibility is also supported by recent methodological studies on the biological underpinnings of multivariate voxel pattern decoding. There are indications that biased voxels for simple stimulus features like orientation or the eye-of-stimulation are distributed across a relatively large scale, because decoding is not very susceptible to spatial smoothing (Op de Beeck, 2010; Shmuel et al., 2010; Swisher et al., 2010). One previous study suggested that orientation decoding was not due to very large scale biases, such as an imbalance in the responses to radial versus tangential orientations (Haynes and Rees, 2005). However, voxel biases may be the result of low spatial frequency harmonics of the high frequency columnar pattern of the cortex (Swisher et al., 2010), or have a more complex relationship to the neuronal populations encompassed by a voxel (Kriegeskorte et al., 2010). Alternatively, biases may reflect stimulus-selective blood vessels (Gardner, 2010; Gardner et al., 2008), which in turn may reflect the anisotropy of the underlying functional architecture of the cortex. In particular, this may be the case for fMRI at 3 T compared to higher field strengths where the fMRI signal is less susceptible to the influence of larger blood vessels (Shmuel et al., 2010), although a direct comparison of voxel pattern decoding at different field strengths has not yet been reported.

The decoding of opposite directions of motion may be difficult even with larger stimuli, because there may be only relatively weak biases in direction preferences evident at the scale of single voxels. Previous MVPA studies varied in their success for decoding the direction of motion from responses in early visual cortex (Brouwer and van Ee, 2007; Kamitani and Tong, 2006). If voxel biases are related (directly or indirectly) to the direction-preference map in visual cortical areas, these weaker biases could be due to the fact that opposite motion directions are encoded by populations within the same orientation columns (Shmuel and Grinvald, 1996). In turn this would mean that the largest difference in voxel biases should exist between orthogonal, not opposite, directions of motion. Recent optical imaging experiments, however, found no direction domains in macaque V1 (Lu et al., 2009), which also suggests that only very weak biases for motion direction may be present in human V1.

We therefore make no claim that the globally coherent context in our experiment enhances motion signals only outside the direct retinotopic regions responding to the stimulus elements. Similarly, our failure to decode the direction of incoherent motion for larger regions of interest from entire visual field quadrants need not mean that decoding for a more effective stimulus would also be at chance. Instead, our study directly pitted globally coherent and incoherent contexts against each other. Our key finding is that decoding was possible for the coherent context but not the incoherent context. This result is not confounded by the question of what biological mechanism underlies direction decoding *per se*, but shows that perceptual grouping enhances the discriminability of motion signals.

Our curve element and the inducers were not only separated in visual space, but were also presented to spatially distinct brain regions. It could nonetheless be argued that because we could only decode when taking voxels corresponding to an entire visual field quadrant, the enhanced decoding for the coherent context was due to ‘spill-over’ of responses from the adjacent visual field quadrant. We believe this is an unlikely explanation because the physical stimulation of the curve quadrant was very similar for the coherent and incoherent contexts. Specifically, inducer elements were always present at a distance of 5.5° from the curve element. At the visual eccentricity employed here, in the early retinotopic areas the population receptive field sizes of individual voxels (Dumoulin and Wandell, 2008; Smith et al., 2001) are much smaller than the inter-element separation in our stimulus (< 1° of visual angle for both V1 and V2). This suggests that voxels in the curve quadrant did not respond directly to any of the inducer elements. Moreover, the globally coherent context was defined by the directions of motion of inducer and curve elements being consistent with a curved path; however, locally the angle between the direction of the curve element and the inducers was identical, merely of the opposite sign. So even if the physical stimulation of the curve quadrant differed for the two contexts, our results must be due to differences in the integration of motion signals, even if this means that locally the receptive fields of voxels encompassed adjacent stimulus elements. Any such interactions between neighbouring elements across the visual field meridians must take into account both the locations and directions (orientations) of the elements. Such a mechanism, whether through local or global interactions, reflects the overall coherence of the stimulus configuration which is precisely the phenomenon we set out to study.

Naturally, in higher extrastriate areas where neurons (and voxels) have larger receptive fields encompassing both the curve element and some or all of the inducer elements the physical difference between the two contexts could account for differences observed in direction decoding. However, interestingly we did not observe above chance decoding of direction of motion in such higher areas.

Improved decoding for direction could be due to a general enhancement of fMRI signals throughout visual cortex. An overall signal increase in an area might also enhance the pattern of voxel biases and thus result in improved decoding performance. We analysed the overall response from regions of interest in the framework of a general linear model used in conventional fMRI analyses. This showed that there were no significant differences in average responses within individual ROIs comparing the coherent and incoherent contexts. If anything, there was a trend for the response to the incoherent context to be greater. This finding is consistent with previous research (Harrison et al., 2007; Murray et al., 2002), but it is incompatible with a stronger response to globally coherent motion. The difference in signal strength for the two contexts probably also accounts for the reliable decoding of context in areas V2, V3, and V4. In accordance with a study combining optical imaging and electrophysiology (Kinoshita et al., 2009), the reduced metabolic activity during coherent motion may reflect the absence of inhibitory neuronal activity, which in turn results in the response facilitation of neurons tuned to the coherent directions of motion.

### Neuronal mechanisms

Perceptual integration may recruit long-range horizontal connections between simple feature detectors in the primary visual cortex that are known to extend several degrees through visual space (Angelucci et al., 2002; Bosking et al., 1997; Chisum et al., 2003; Fitzpatrick, 2000). The profile of these connections has been well-described and they are likely to play a role in various contextual interactions that modulate neuronal responses (Chisum et al., 2003; Kapadia et al., 2000). They link neurons with similar orientation preferences (Bosking et al., 1997; Gilbert and Wiesel, 1989). However, they are organized primarily along the axis of the preferred orientation of a neuron. They would thus facilitate the activity evoked by co-circular edges, which argues against their involvement in our motion stimulus where elements are oriented to be near parallel to one another. Also, at the eccentricity we used here (between 4.1° and 8.4°), the individual grating elements are placed close to the spatial extent of these lateral connections (Angelucci et al., 2002), which does not rule their involvement, although it makes other factors more likely.

Neurons in higher visual cortical areas with large receptive fields pool the signals from individual stimulus elements. Neurons selective for moving contours and shapes will respond more strongly to the coherent context. We found that it was possible to use voxel patterns from areas V2, V3, and V4 to decode between the coherent and incoherent contexts. It is therefore possible that feedback from higher extrastriate cortex to earlier retinotopic cortex enhances the response of neuronal populations selective for the current direction of the moving stimulus (either directly or indirectly by reducing inhibition between populations with different direction-selectivity).

One previous study employed dynamic causal modelling (Friston et al., 2003) to investigate the role of feedforward and feedback connections between V1 and V5/MT during apparent motion using a stimulus configuration very similar to the one we employed in the present study (Sterzer et al., 2006). During the percept of apparent motion, feedback from V5/MT to the retinotopic location of the apparent motion in V1 was enhanced (in the absence of physical stimulation). Moreover, in behavioural studies, drifting Gabor stimuli also result in an illusory percept that may be related to apparent motion: the Gabor is perceived as displaced from its veridical location along the axis of motion (Bex et al., 2001; De Valois and De Valois, 1991). We speculate that the percept of globally coherent motion associated with our stimuli also involves feedback from higher extrastriate cortex and that it differed between the coherent and incoherent contexts. This could explain why we observed better decoding when taking into account voxels that arguably received no direct physical stimulation, and may represent a promising direction for future study.

### Upper versus lower hemifield asymmetry

We observed differences in decoding accuracy comparing upper and lower visual fields. Decoding was only significantly different for the coherent and incoherent contexts when the global context was in the lower-right quadrant but not when it was in the upper-left quadrant. Similarly, only for the lower-right quadrant was decoding accuracy for direction of motion above chance. We therefore surmise that the effect of globally coherent motion may be stronger for the lower visual field. Indeed, there have been reports of an asymmetry between the upper and lower visual fields for perceptual integration (Rubin et al., 1996). This may be because vision in the lower visual field is required predominantly for detecting and identifying objects, such as prey or predators, and to ascertain the topography of the ground to navigate steps and steep edges.

### Grouping and perceived speed

In behavioural experiments outside the scanner environment we examined the effect of the globally coherent and incoherent contexts we employed in the main fMRI experiment had on the perceived speed of the Gabor elements. One previous study reported that when several grating elements are moving in the same direction speed discrimination is worse than when a single grating is shown in isolation suggesting that grouping interferes with speed judgements (Verghese and Stone, 1996). While most of our observers showed worse speed judgements for the contextual stimuli containing several scattered elements relative to stimuli with only a single element, we found no difference in this effect between coherent and incoherent contexts. Further, the direction of the effect was not consistent across participants, as for some participants speed was perceived as faster while for others it was perceived to be slower than the veridical speed. We therefore surmise that previously reported effects of grouping on perceived speed (Verghese and McKee, 2006; Verghese and Stone, 1996) are unrelated to the enhanced discriminability of motion signals in visual cortex that we report here.

## Conclusion

Here we showed that coherent moving stimuli alluding to the presence of a global object resulted in reliable decoding of the direction of motion, as long as the stimulus was in the lower visual field. This indicates that early visual cortex contains information about the presence of moving objects, not merely their component motion features. An enhanced representation of the direction of motion may allow the visual system to infer the trajectory of movement across the gaps between individual stimulus features and thus play a role in perceptual grouping when local elements are interpreted as being part of a coherent whole.
