# Supplementary material for: Decoding of coherent but not incoherent motion signals in early dorsal visual cortex
Source: Neuroimage. 2011 May 15;56(2-10):688–98. doi: 10.1016/j.neuroimage.2010.04.011 (PMC3084455; doi:10.1016/j.neuroimage.2010.04.011)
Supplement: Supplementary file 1 [file mmc1.doc]

**Supplementary materials**

*Ensuring accurate fixation*

Our results for areas containing maps of separate visual field quadrants crucially depend on participants maintaining accurate fixation throughout the experiment. In order to assure fixation and arousal during the scanning sessions, participants were asked to monitor the fixation cross for brief changes in luminance. Overall performance on this task was high but not at ceiling (78.1%) and false alarm rate was very low (0.6%).

We also measured eye movements in two participants (CP and AS) during scanning with a sampling rate of 60 Hz. After removal of periods of signal loss and eye blinks we compared the mean horizontal and vertical eye positions as well as the pupil diameter with a participant-specific repeated measures ANOVA. We found that both participants showed good fixation throughout the experiment with no differences between stimulus conditions, neither for horizontal eye position (CP: F(3,15)=1.47, p=0.237; AS: F(3,15)<1, p=0.889), vertical eye position (CP: F(3,15)=1.29, p=0.290; AS: F(3,15)<1, p=0.928) nor pupil diameter (CP: F(3,15)<1, p=0.477; AS: F(3,15)<1, p=0.802).

*Stimulus examples*

These movies show examples of those used in the fMRI experiment. Gabors could either be arranged to be in a coherent or incoherent global context. In separate blocks elements would drift either anti-clockwise or clockwise (but here the two directions are shown in immediate succession). In separate scanning sessions the curve either traversed the lower-right or the upper-left visual field. In these example movies it traversed the lower-right visual field.

**Supplementary figure legends**

**Figure S1.** Flat maps from a representative participant. A. The response to all stimuli in the main experiment relative to blocks of fixation is plotted at a relaxed statistical threshold (p<0.05, uncorrected). B-C. Voxels in V1d (representing the curve quadrant) showing a moderately significant bias for direction of motion (p<0.1, uncorrected) in the coherent context (B) and the incoherent context (C). The dotted black lines indicate the boundaries of visual areas. The yellow dotted line denotes the location of the ROI defined by the ‘element localizer’. The mean decoding accuracy (± standard error of the mean) for direction of motion in V1d across all instances of cross-validation is denoted above each map.

**Figure S2.** Psychometric curves in the behavioural experiment for each of the four participants (in rows). We plotted the proportion of trials in which the test stimulus was perceived as faster than the reference as a function of the veridical ratio between test and reference speed (in logarithmic units). Thus a rightward shift of the curve would indicate that participants saw the stimulus as moving more slowly than the reference stimulus. Data are shown either for pooling data from the two possible positions of the curve quadrant (left), or separately for when the curve quadrant was the lower-right (middle) or upper-left quadrant (right). Symbols indicate raw data; the solid lines are the best fitting models. Blue: coherent context. Red: incoherent context. Green: control (single element in both reference as well as test stimulus).
