# Supplementary figures and images for: Decoding of coherent but not incoherent motion signals in early dorsal visual cortex
Source: Neuroimage. 2011 May 15;56(2-10):688–98. doi: 10.1016/j.neuroimage.2010.04.011 (PMC3084455; doi:10.1016/j.neuroimage.2010.04.011)

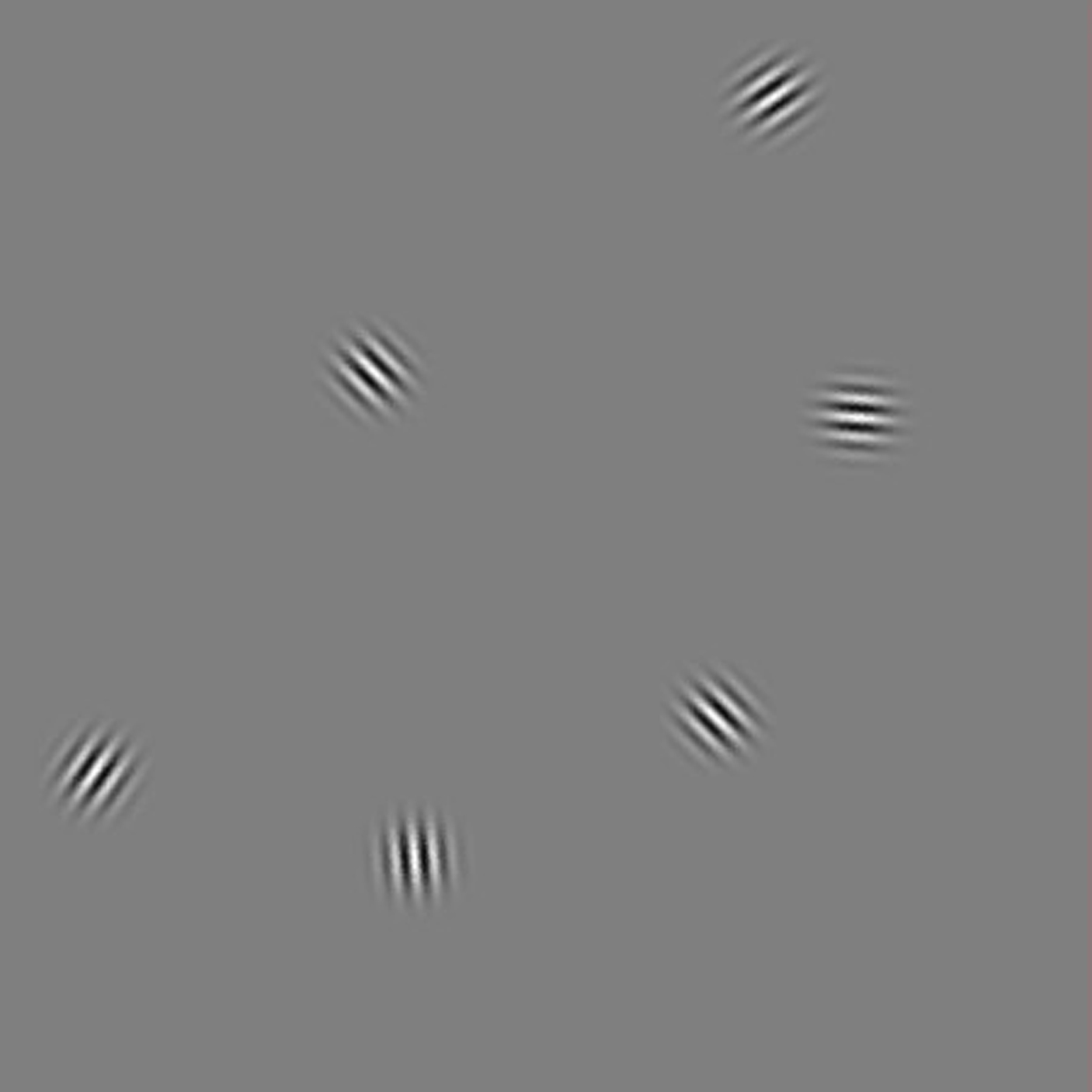

Supplement: Supplementary file 2 [file mmc2.jpg]
